# Direct growth of ultra-long platinum nanolawns on a semiconductor photocatalyst

**DOI:** 10.1186/1556-276X-6-380

**Published:** 2011-05-13

**Authors:** Yu-Lin Shen, Shih-Yun Chen, Jenn-Ming Song, Tzu-Kang Chin, Chu-Hsuan Lin, In-Gann Chen

**Affiliations:** 1 Graduate Institute of Engineering, National Taiwan University of Science and Technology, Taipei 106, Taiwan; 2 Department of Materials Science and Engineering, National Taiwan University of Science and Technology, Taipei 106, Taiwan; 3 Department of Materials Science and Engineering, National Dong Hwa University, Hualien 974, Taiwan; 4 Graduate Institute of Opto-electronic Engineering, National Dong Hwa University, Hualien 974, Taiwan; 5 Department of Materials Science and Engineering, National Cheng Kung University, Tainan 701, Taiwan

## Abstract

A template- and surfactant-free process, thermally assisted photoreduction, is developed to prepare vertically grown ultra-long Pt nanowires (NWs) (about 30-40 nm in diameter, 5-6 μm in length, and up to 80 NWs/100 μm^2 ^in the wire density) on TiO_2 _coated substrates, including Si wafers and carbon fibers, with the assistance of the photocatalytic ability and semiconductor characteristics of TiO_2_. A remarkable aspect ratio of up to 200 can be achieved. TEM analytical results suggest that the Pt NWs are single-crystalline with a preferred 〈111〉 growth direction. The precursor adopted and the heat treatment conditions are crucial for the yield of NWs. The photoelectrons supplied by TiO_2 _gives rise to the formation of nano-sized Pt nuclei from salt melt or solution. The subsequent growth of NWs is supported by the thermal electrons which also generated from TiO_2 _during the post thermal treatment. The interactions between the ions and the electrons in the Pt/TiO_2 _junction are discussed in this study.

## Introduction

Platinum has become a crucial material due to its outstanding catalystic characters in fuel-cell technology, hydrogenation reaction, three-way automobile catalytic conversion and gas sensing [1-4]. A great deal of effort has been devoted to the synthesis of one-dimensional Pt nanostructures, however, it still remains a grand challenge to synthesize long and oriented single-crystalline Pt nanowires (NWs). Table 1 summarizes the relevant reports concerning template-free chemical means for preparing Pt NWs. Xia et al. [5-7] have demonstrated the synthesis of single crystalline Pt NWs on polymeric, ceramic or metallic substrate by a polyol process, combined with a trace addition of an iron species (Fe^2+ ^or Fe^3+^) and poly(vinylpyrrolidone) (PVP) as the surfactant. Cetyltrimethylammonium bromide (CTAB) has also been applied in the reduction of Pt ions to Pt NWs [8,9]. Moreover, using neither templates nor surfactants, HCOOH [10-13] and vitamin B_2 _[14] have been suggested, respectively, to act as the reductant agents in the chemical routes for the synthesis of Pt NWs. Through the aforementioned processes, the Pt NWs produced are extremely fine (<10 nm in diameter) but exhibit a limit in length of about 200 nm so that their aspect ratios do not exceed 50.

**Table 1:** A summary of prior works on the template-less synthesis of Pt NWs

Method	Orientation	Surfactant (S) or reductant (R)	Diameter (nm)	Length	Crystallization	Precursor	Authors
Chemical reduction	〈111〉	PVP (S)	~5	~200 nm	Single-crystalline	H_2_PtCl_6_	Xia et al. [5-7]
Chemical reduction	-	CTAB (S)	2.2	Networks	Polycrystalline	K_2_PtCl_4_	Song et al. [8]
Photochemical reduction	〈111〉	CTAB (S)	3-4	20-60 nm	Single-crystalline	K_2_PtCl_4_	Krishnaswamy et al. [9]
Chemical reduction	〈111〉	HCOOH (R)	4	100-200 nm	Single-crystalline	H_2_PtCl_6_	Sun et al. [10-13]
Chemical reduction	-	Vitamin B_2 _(R)	10	Several tens of nm	Polycrystalline	Pt metal salt	Nadagouda et al. [14]
Thermal-assisted photoreduction	〈111〉	-	32	6 μm	Single-crystalline	Na_2_Pt(OH)_6_	Our group

*PVP *(poly(vinylpyrrolidone)), *CTAB *(cetyltrimethylammonium bromide).

It is well known that TiO_2 _is an excellent photocatalyst under exposure to ultraviolet (UV) light. According to the Honda-Fujishima effect [15], electrons and holes on the surface of TiO_2 _films can be activated by UV light, which enables the reduction of metallic ions from the solution. The photoreduction process of metallic ions (M^*+*^) can be expressed briefly as follows,

Based on this, a recently developed process, thermally assisted photoreduction (TAP), has been applied to prepare metallic NWs via the photoreduction of metallic ions on the surface of thin-film TiO_2 _under certain irradiating and heating conditions [16,17]. However, it has so far not been possible to produce Pt NWs with the commonly used precursor, H_2_PtCl_6_, which was ascribed to the high charge number of Pt ions.

By extending the selection of the precursors, a modified route for the synthesis of Pt NWs is proposed in this study. In addition to Si wafers, carbon cloths are also chosen as the substrate for investigation, since nanostructured Pt-TiO_2 _on carbon fibers or nanotubes has been suggested as excellent electrocatalysts for direct enthanol fuel cells [18,19]. Considering the mechanism for forming the NWs was still elusive, this study also discusses the growth of Pt NWs from the view points of the transfer of ions and electrons, as well as their interactions.

## Experimental details

### Thin film preparation

**Figure 1:**
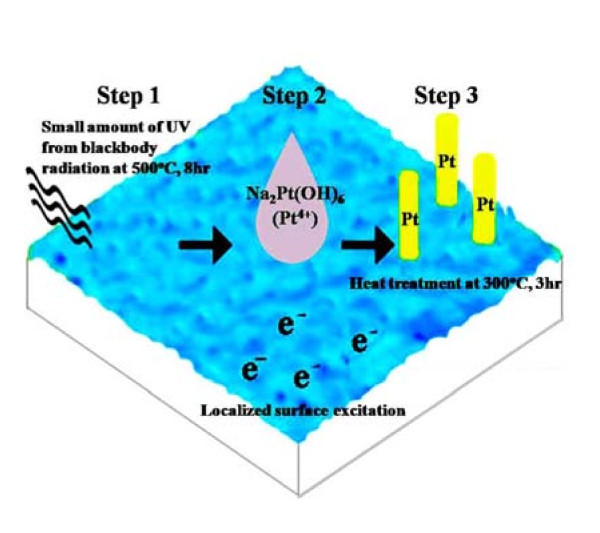
**Schematic illusion of the main steps for forming Pt NWs by TAP**.

**Figure 2:**
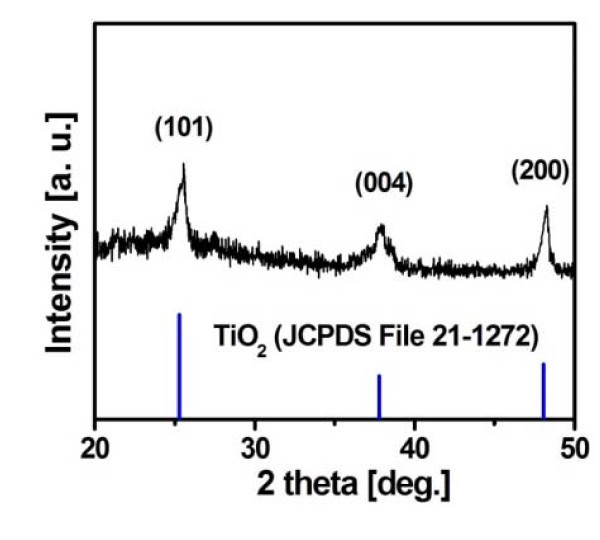
**XRD pattern of the TiO**_**2 **_**thin film annealed at 500°C for 8 h **.

To make a gel coating film, TiO_2 _was deposited by dipping Si wafers and carbon cloths into the sol and then only the wafers were spun at 1000 rpm for 30 s. The solution used was prepared with isopropylalcohol (IPA):titanium isopropoxide (TTIP):hydrogen chloride (HCl) with a volume ratio of 170:12:0.4 and stirred for 10 min before aging at room temperature (20°C) for 2 days. The as-synthesized TiO_2 _films were annealed at 500°C for 8 h in an oxygen atmosphere to obtain well-crystallized anatase TiO_2 _(step 1 in Figure 1), which could be identified by the grazing-angle X-ray diffraction (XRD) pattern shown in Figure 2.

### NWs synthesis

As indicated by the step 2 in Figure 1, Na_2_Pt(OH)_6 _was selected as the precursor. Fifteen microliter of 0.05 M aqueous salt solution was dropped on the TiO_2 _coated substrates. Afterward the samples were isothermally heated at 300°C for 3 h in air by an infrared (IR) furnace (the same heating conditions as adopted in previous studies [16,17]), followed by a furnace-cooling to the ambient temperature (namely the post thermal treatment, step 3 in Figure 1). For comparison, the commonly used precursor, H_2_PtCl_6_, was also adopted. To clarify how the state of the precursor affects the yield of NWs, the precursor was also applied in the form of powders.

### NWs characterizations

The structure and phase of the NWs were characterized using a transmission electron microscope (FEI-TEM, Philips Technai G2) with an accelerating voltage of 200 kV and also an grazing incidence X-ray diffraction meter (GIXRD, Rigaku D/MAX2500) (incidence angle of 0.5°) with graphite monochromatic Cu Kα radiation (λ = 0.15418 nm) at a scanning rate of 2° per minute from 20° to 50°. The yield (i.e. the wire number per 100 μm^2^) and dimensions of NWs were measured using a scanning electron microscope (SEM, JEOL JSM-6700) and Scion Image 4.0.2 image analysis software. Each data was the average of 100 observations.

## Results and discussion

**Figure 3:**
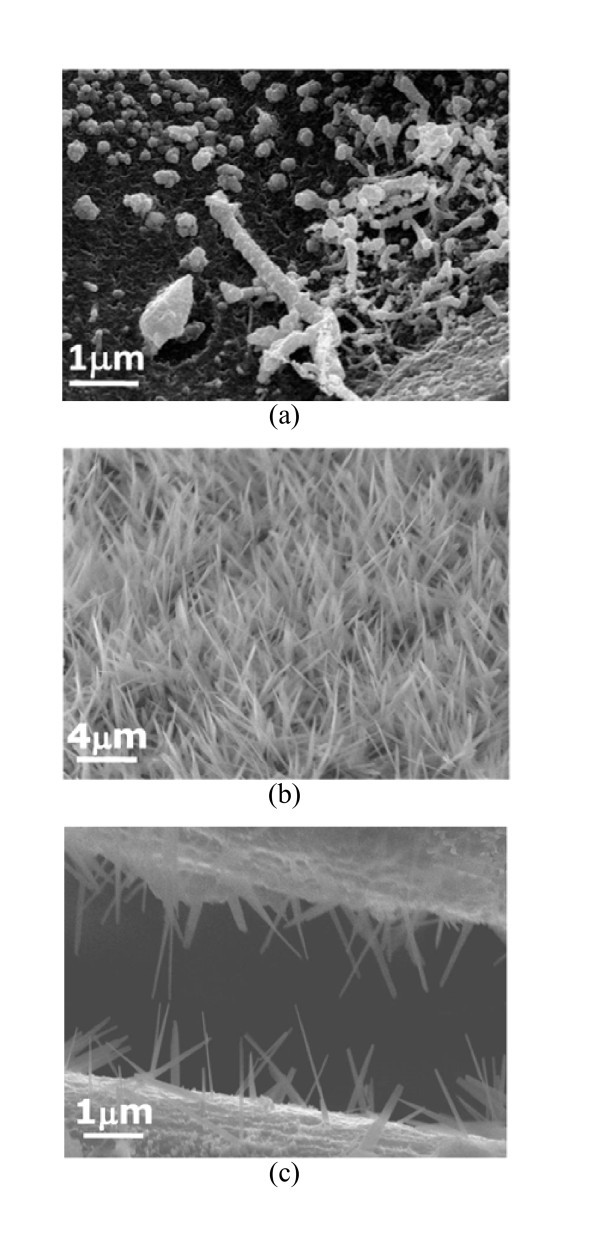
**Nano- or micron-structures obtained from different Pt salts on TiO**_**2 **_**coated substrates subjected to heating at 300°C**: **(a) **H_2_PtCl_6 _on Si wafer, **(b) **Na_2_Pt(OH)_6 _on Si wafer and **(c) **Na_2_Pt(OH)_6 _on carbon fibers.

After heating the salt solutions at 300°C for 3 h, the SEM image shown in Figure 3a illustrates that the commonly used precursor, H_2_PtCl_6_, still remained untransformed and was with nodular or worm-like appearance. For Na_2_Pt(OH)_6 _(Figure 3b), all the Pt salt was transformed into Pt NWs in large quantities, with an average diameter of 34 nm and remarkable length of about 6 μm (a superb aspect ratio of up to 200, which is the greatest value reported so far, among Pt NWs synthesized by various template-less and surfactant-free methods), grew vertically on the TiO_2 _coated Si substrate. No byproducts were found and thus no purificatory procedures are needed. Ultra-long Pt NWs can also be prepared on other substrate materials coated with TiO_2_. Figure 3c displays Pt NWs grown radially on carbon fibers. The above results manifest that the selection of the precursor was crucial. Given that the melting point of H_2_PtCl_6 _is 60°C [20], it can be deduced that H_2_PtCl_6 _melted and re-solidified during the thermal treatment. The transformation of Na_2_Pt(OH)_6 _into Pt NWs was associated with the relatively lower electronegativity of the OH group (3.02) [21] compared to Cl (3.16), which may cause the metal ions to be reduced easily, thus allow the growth of NWs.

**Figure 4:**
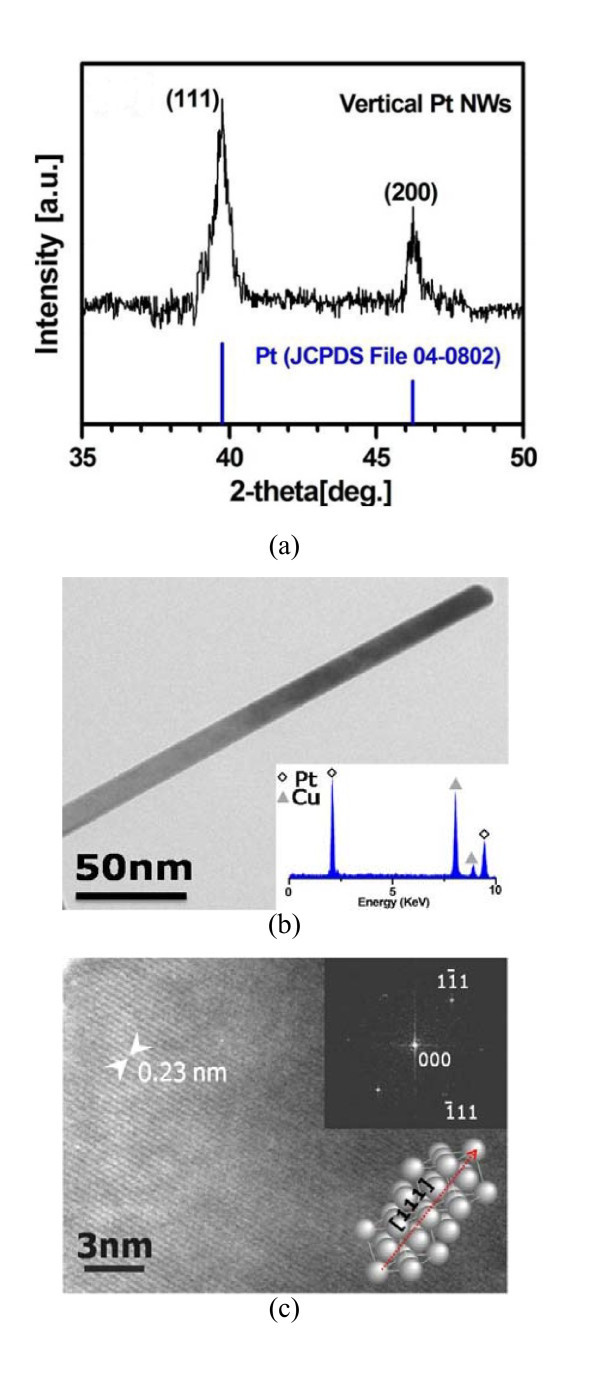
**Ultra-long Pt NWs and their properties**: **(a) **the XRD pattern, **(b) **the TEM image and EDS results (the Cu signal comes from the Cu grids) and **(c) **the HRTEM lattice image of the NW in **(b) **and the corresponding electron diffraction patterns by fast Fourier transform, as well as a illustration showing a FCC crystal with the growth direction along [111].

The XRD pattern and EDS spectrum (Figure 4a,b) demonstrate that the NWs thus produced were pure Pt with an FCC structure without any detectable impurity. For a better understanding of the structure of Pt NWs, a high-resolution transmission electron microscope (HRTEM) image recorded from a Pt NW (Figure 4c) depicts the lattice spacing between the {111} planes of 0.23 nm, which was in agreement with the value in a bulk Pt crystal, suggesting the growth direction of the Pt NWs was along 〈111〉 axes. The inserted electron diffraction pattern constructed by fast Fourier transform (FFT) also verifies this preferred growth direction.

**Figure 5:**
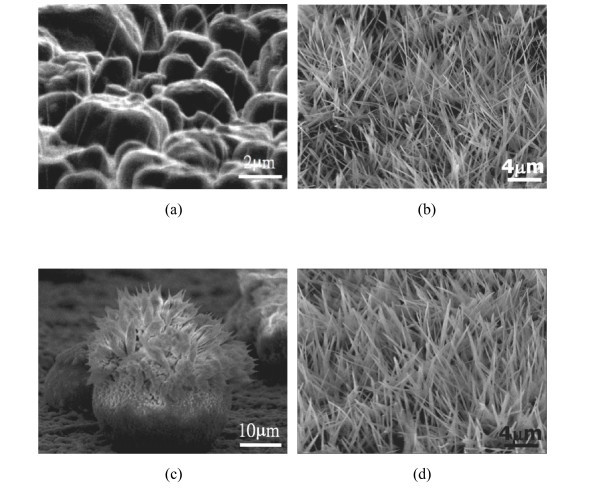
**The reaction products on the TiO**_**2 **_**coated substrate subjected to TAP process**: **(a) **the solution samples heated at 140°C, **(b) **the solution samples heated at 160°C, **(c) **the powder samples heated at 140°C and **(d) **the powder samples heated at 160°C.

**Figure 6:**
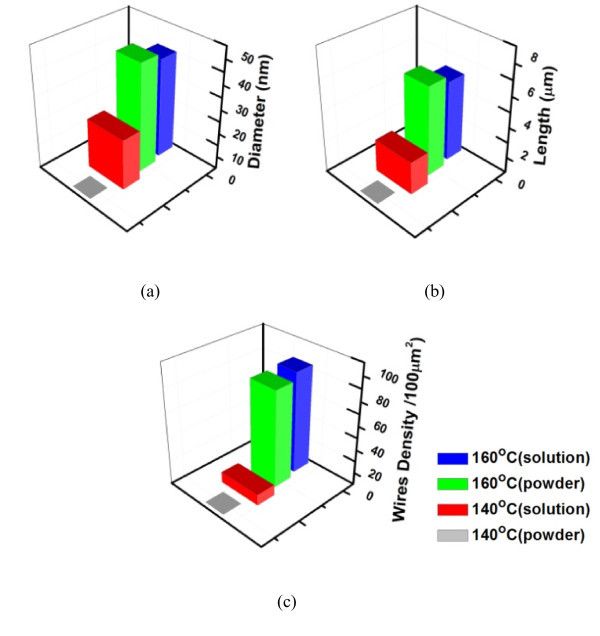
**Histograms of the quantitative data of Pt NWs under different growth conditions**: **(a) **diameter, **(b) **wire length and **(c) **wire density (the yield of NWs).

To clarify how the state of the precursor affects the yield of NWs, Na_2_Pt(OH)_6 _powders and aqueous solution were placed or dropped on the TiO_2 _coated Si wafers, respectively, and isothermally treated at chosen temperatures for 3 h in air by an IR furnace. According to the melting point of Na_2_Pt(OH)_6_, 150°C [20], the thermal treatment was held at 140°C to keep the salt in solid state or 160°C to turn it into liquid. The SEM images in Figure 5 show the morphologies of the samples after the TAP routes. For the solution samples after being thermal-treated at 140°C for 3 h (Figure 5a), chunky Na_2_Pt(OH)_6 _nodules and very few Pt NWs located in between them could be observed. Interestingly, when the holding temperature was raised to 160°C, the Pt salt solution was transformed into a plenty of Pt NWs with the average diameter of 40.9 nm and length of about 5.2 μm (Figures 5b and 6a,b), which were slightly shorter and thicker than those synthesized at 300°C. Similar results were observed if the aqueous solution was replaced by powders. As illustrated in Figure 5c, the powders of Na_2_Pt(OH)_6 _remained without forming any NWs subsequent to the post thermal treatment at 140°C. Remarkably, after isothermal heating at 160°C, Na_2_Pt(OH)_6 _powders were transformed to Pt nanolawns, with average wire diameter and length of 44.6 nm and 5.8 μm, respectively (Figures 5d and 6a,b).

**Figure 7:**
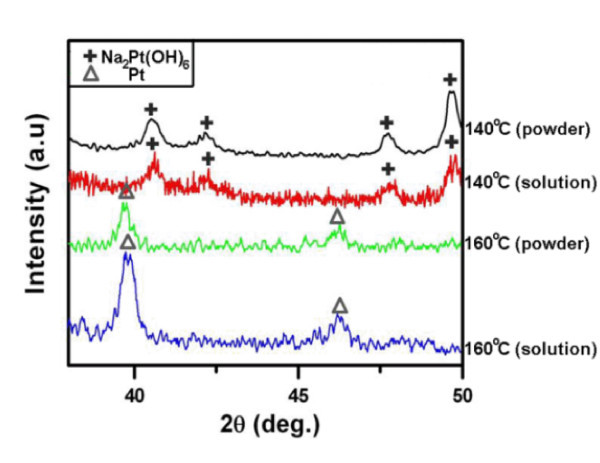
**The XRD patterns of the samples subjected to thermal treatment at various temperatures**.

Quantative data given in Figure 6c reveal that a large yield of NWs of up to 80 NWs/100 μm^2 ^could be obtained for both solution and powder samples subjected to thermal treatment at 160°C, i.e. 10°C higher than the melting point of Na_2_Pt(OH)_6_. Heating at 140°C, NWs were barely observed in the Na_2_Pt(OH)_6 _aqueous solution samples; and further, they were absent in the powder samples. The XRD patterns shown in Figure 7 support the above observations.

The state of Pt salts indeed caused a great difference in the NW yield. With respect to the solution samples at 140°C, water soon evaporized upon heating and thus Na_2_Pt(OH)_6 _re-precipitated as massive nodules. The evidence that a very small number of Pt NWs could be found in the solution samples heated at 140°C implies that dissociated Na_2_Pt(OH)_6 _in water was still capable of forming NWs, however, the reaction time was quite limited because water vaporized and depleted fast. At 160°C, Na_2_Pt(OH)_6 _in both solution and powder samples melted and transformed into NWs effectively. Accordingly, it is reasonable to infer that free ions in an electrolyte, especially the molten salt, are important for the resultant yield of Pt NWs.

**Figure 8:**
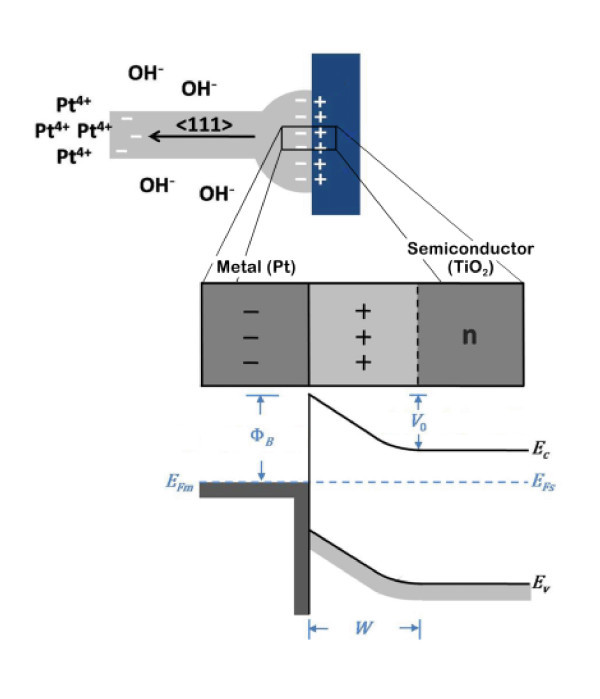
**Proposed model for the growth of Pt NWs during the TAP process**.

It has been demonstrated that metallic ions can be reduced to form nano-sized nuclei on the surface of photocatalytic TiO_2 _[22]. In our case, once the Pt^4+ ^ions gained photoelectrons and the elemental Pt started to precipite on the surface of TiO_2_, a Schottky barrier formed since the work function of Pt (5.65 eV) [23] is larger than TiO_2 _(4.2 eV) [24], a n-type semiconductor according to the results of Hall measurement listed in Table 2. As sketched in Figure 8, in order to align Fermi levels for the metal (*E*_Fm_) and semiconductor (*E*_Fs_) at equilibrium, electrons move to the metal and then the positive charge due to ionized donor ions within *W*, the depletion region adjacent to the junction, matches the negative charge on the metal [25]. The equilibrium contact potential *V*_o _(so-called built-in potential barrier, the difference in work functions between Pt and TiO_2_) prevents further net electron diffusion from the semiconductor conduction band into the metal. On the other side of the junction, *Φ*_*B *_is the potential barrier for backward electron flow from the metal to the semiconductor.

**Table 2:** Electrical properties of TiO_2 _obtained from the Hall measurement

Type	**Carrier concentration (cm**^**-3**^**)**	**Mobility (cm**^**2**^**/V s)**	Resistivity (Ω cm)
n	1.89 × 10^17^	53	6.23 × 10^-1^

It is suggested that during the subsequent thermal treatment, thermal electrons generated in TiO_2 _can supply the follow-up reduction of Pt ions to grow NWs from the nano-sized nuclei. The formula for calculating the number of thermal electrons (*n*) in a semiconductor as a function of temperature is shown below [26]:

where *k *is Boltzmann's constant, *h *is Planck's constant, *m*_e _and *m*_h _are the effective mass of electron and hole, *T *is calculated in Kelvin and *E*_g _is the band gap. It can be deduced that the great standard reduction potential of Pt^4+ ^(1.15 V) can overcome the potential barrier, *V*_o_, and gave rise to a net electron flow for further reduction of the metallic ions. In order to maintain the charge neutrality at the Pt/TiO_2 _interface, hydroxide ions (OH^-^) in the salt melt or aqueous solution were able to balance the positive charge on the TiO_2 _side, as illustrated in Figure 8. As a result, the thermal electrons attracted by Pt^4+ ^were allowed to cross the interface and diffuse to the growing front of Pt nuclei. Consequently, the reduced Pt atoms accumulated and stacked on the closest packed facet to form 〈111〉-oriented one-dimensional nanostructure.

## Conclusions

By means of the TAP process, vertically grown ultra-long Pt NWs with the remarkable aspect ratio of up to 200 can be obtained on the TiO_2 _coated substrate in large quantities, without the additional assistance of surfactants, templates and seeding. The Pt NWs are single-crystalline with preferred growth direction along 〈111〉. The selection and the state of the precursor are crucial for the yield of NWs. The nucleation and the growth of NWs are, respectively, supported by the photoelectrons and thermal electrons subsequently excited from TiO_2_. A model describing the interactions between the ions (Pt^4+ ^and OH^-^) and the electrons (photo- and thermal electrons) in the Pt/TiO_2 _junction was proposed and can be adopted to design the template-less fabrication process of ultra-long metallic NWs for further application.

## Abbreviations

CTAB: cetyltrimethylammonium bromide; FFT: fast Fourier transform; HRTEM: high-resolution transmission electron microscope; HCl: hydrogen chloride; IPA: isopropylalcohol; IR: infrared; NWs: nanowires; PVP: poly(vinylpyrrolidone); TAP: thermally assisted photoreduction; TTIP: titanium isopropoxide; TEM: transmission electron microscope; UV: ultraviolet; XRD: X-ray diffraction.

## Competing interests

The authors declare that they have no competing interests.

## Authors' contributions

YLS carried out the main part of synthetic and analytical works, participated in the sequence alignment and drafted the manuscript. TKC participated in the synthetic experiments. SYC and CHL participated in the discussion of the growth mechanism. JMS participated in the design of the study, draft preparation and coordination. IGC conceived of the study and participated in its design. All authors read and approved the final manuscript.
